# Proactive end-of-life conversations in residential care homes: a qualitative interview study exploring residents’ and family members’ experiences

**DOI:** 10.1186/s12877-025-05916-7

**Published:** 2025-04-25

**Authors:** Åsa Mikaelsson, Lars E. Eriksson, Terese Stenfors, Ida Goliath

**Affiliations:** 1 Department of Neurobiology, Care Sciences and Society, Karolinska Institutet, Huddinge, Sweden; 2 School of Health and Psychological Sciences, City, University of London, London, UK; 3 Department of Learning, Informatics, Management and Ethics, Karolinska Institutet, Solna, Stockholm, Sweden; 4 Stockholm Gerontology Research Center, Stockholm, Sweden; 5 Medical Unit of Infectious Diseases, Karolinska University Hospital, Huddinge, Sweden

**Keywords:** Residential care home, End-of-life conversations, Advance care planning, Qualitative research, Family support, Person centred care, Dementia

## Abstract

**Background:**

Due to population aging, residential care homes are increasingly providing end-of-life care for residents with multiple chronic illnesses and cognitive decline. Proactive end-of-life communication, a component of Advance Care Planning, has been suggested as a means of providing high-quality care aligned with residents’ preferences and supporting involved family members. Despite growing knowledge about the benefits of early communication concerning end-of-life care preferences, such conversations are still rare in the context of residential care homes, and little is known about how they are perceived by residents and family members. The aim of this study is to explore the outcomes experienced by residents and family members who have participated in proactive end-of-life conversations in residential care homes.

**Methods:**

This qualitative study is embedded within a participatory action research project implementing proactive end-of-life conversations in five Swedish residential care homes, using a conversation tool. In this study we performed 18 interviews with eleven residents and eight family members after they had participated in staff initiated proactive end-of-life conversations. Data were analyzed using interpretive description.

**Results:**

Residents and family members experienced several outcomes of proactive end-of-life conversations presented in three closely interconnected themes: (1) Enabling open communication, (2) Creating space for knowledge exchange, and (3) Contributing to feelings of confidence and relationship building.

**Conclusions:**

Proactive end-of-life conversations generated several beneficial outcomes for residents and family members, including those with cognitive decline. The study demonstrated that the conversations may strengthen person-centered care and family support in this context. Based on these findings, proactive end-of-life conversations have the potential for use by residential care home staff.

**Supplementary Information:**

The online version contains supplementary material available at 10.1186/s12877-025-05916-7.

## Background

Population aging implies a growing need worldwide for extended support and care at the end-of-life (EoL), and residential care homes (RCHs) [1] are common providers of such care [2, 3]. Older persons who move into RCHs often have multiple chronic health issues [4], frailty, and experience gradual deterioration [5], including cognitive decline [6–8]. This results in extensive care needs in the final phase of life [9], requiring elder care systems that are forward-thinking. The World Health Organization emphasizes the necessary transitioning from reactive disease-centered models to proactive health-based models that focus on a person’s capacity in old age [10]. Enabling people to express their care preferences is essential to the provision of value concordant care; this is also true for those with cognitive decline. The right to receive appropriate support to maintain the greatest possible participation has also been declared as a human right [11]. Family members often play a crucial role in caring for and supporting older people, including after their admission to a long-term care facility [12]. This is particularly the case when RCH residents experience cognitive limitations [13, 14].

The involvement of residents and family members in proactive communication about the EoL is considered a key factor for promoting high quality EoL care in RCHs [15, 16]. This promotes the provision of care consistent with residents’ wishes, reduces unnecessary hospitalization and treatment [17], and enables the involvement of family members in EoL care [15]. EoL conversations are a component of advance care planning (ACP), an umbrella term defined as a process of discussing and documenting EoL care goals and preferences with patients, family members and care providers [18]. ACP has evolved over the decades from an initial focus on legal transactions with people having decisional capacity, to a broader continuum of repeated communication about EoL care values and preferences across the lifespan [19]. One critique is the great variability in the way researchers and professionals approach and conceptualize ACP, making the evaluation of outcomes challenging [20, 21]. Nevertheless, ACP has been associated with increased quality of care and patient satisfaction [22], improved end-of-life care quality [17, 23, 24], increased caregiver satisfaction with quality of care and communication [25, 26], as well as a reduction in the number of unnecessary and unwanted care procedures [17, 23] and hospitalization episodes for frail older persons [27]. In addition, a lack of quality EoL communication has been raised as a common cause of family members’ dissatisfaction with EoL care [28]. To further address the EoL communication needs of people with cognitive decline, Van der Steen et al. [29] recently proposed a framework emphasizing ACP as a continuous, supportive, and adaptive process to promote the inclusiveness of persons with dementia and their family members. In addition, it has been suggested that such conversations should be initiated early in the care trajectory to better enable residents with cognitive decline to participate [15, 30].

While it is widely recognized that EoL conversations are essential in the RCH context [31–34], and several initiatives to implement and explore EoL communication in RCHs have been conducted [35–39], such conversations are still rare and there is limited knowledge about how they are perceived by residents and family members [40–42]. This is particularly the case for residents with cognitive impairment [38].

## Methods

### Aim

The aim of this study is to explore the outcomes experienced by residents and family members who have participated in proactive EoL conversations in RCHs.

### Study design

This qualitative study is part of a larger multi-case participatory action research endeavor to implement proactive EoL conversations across five Swedish RCHs. In the current study, we applied an interpretive description approach, a flexible methodology grounded in the epistemological framework of applied sciences that has been widely used in nursing research for exploring phenomena of relevance for the clinical context [43–45]. This study followed the Consolidated Criteria for Reporting Qualitative Studies (COREQ) guidelines [46].

### Setting

In Sweden, about a third of all deaths take place in RCHs [47]. Elder care is publicly funded, with self-governing municipalities being responsible for care provision while state authorities oversee regulation and monitoring. Living in a RCH includes 24-hour access to staff assisting with daily life activities, e.g., help with hygiene routines, eating, cleaning and administration of medication [48]. The most common staff on-site in RCHs are nurse assistants, followed by registered nurses, physiotherapists, and occupational therapists, while physicians are available at set hours or by phone [49]. An estimated two-thirds of all Swedish RCH residents are believed to have cognitive impairment, a condition often considered underdiagnosed within these facilities [50, 51].

### Procedure of proactive eol conversations

In this study we use the term proactive EoL conversations for voluntary structured staff-initiated conversations with RCH residents and/or their family members, focusing on the EoL values and preferences of the residents. This study is based on follow up interviews subsequent to 17 proactive EoL conversations performed in five RCHs (Table 1). During the conversations, a research-based conversation tool was used, in the form of a card deck with statements promoting reflection on EoL values and preferences [52]. The conversation tool, in Swedish named the DöBra^1^ cards, consists of 37 statements covering physical, practical, existential, and social issues, e.g., “To be free of pain”, “To be able to talk about what scares me,” “To have my financial affairs in order”, “To have close friends near”, etc., and two “wild cards” to identify other matters of importance to the person [53]. Prior to this study, the conversation tool was used in a two-session staff training program based on an experiential learning approach, promoting self-reflection and knowledge exchange, with the aim of preparing staff for conducting proactive EoL conversations. The tool was then used by staff during proactive EoL conversations. According to the instructions accompanying the tool, each card is to be sorted into one of three piles: very important, important, and less important. The cards in the most important pile are then to be ranked according to priority. However, it is worth noting that the procedure should be seen as a process enabling reflection and communication on the person’s values and priorities [53] so, for this study, the staff were encouraged to adapt the use of the cards to residents’ and/or family members’ abilities. Although we were informed that proactive EoL conversations were performed in all five RCHs we have no data describing the number, duration or constellations of the conversations.

### Recruitment

We used purposive sampling to recruit participants who had participated in proactive EoL conversations as part of the main multi-case participatory action research endeavor [43]. Sample size was guided by information power [54] and based on the study’s aim and expectations of information in the sample. We initially estimated the sample size to 15–20 individuals, with roughly an equal number of residents and family members. During the data collection, a larger number of residents were included, as several had cognitive difficulties, which made data less articulated and information rich. Staff were encouraged to ask residents and family members after the proactive EoL conversations if they were willing to be contacted by a researcher and provided contact details if they agreed.

Twelve residents and ten family members who had participated in proactive EoL conversations received written and verbal information about the study from RCH staff who then provided the first author (ÅM) with contact details of those who had agreed to be contacted. One of the residents declined participation due to deteriorating health and three family members declined participation due to the residents’ deteriorating health, residents’ recent death, or their own time constraints.

### Participants

Eleven residents and eight family members consented to participate in this interview study (Table 1). The residents, seven females and four males, were aged between 76 and 98 years (mean = 88). Three had been diagnosed with dementia, although nine of the eleven participating residents displayed signs of cognitive decline during the interviews, e.g., not remembering events, repeating stories, or having difficulty finding words. Eight family members participated in seven interviews; two sisters participated in a joint interview, and two sisters participated in individual interviews. One family member was related to a participating resident. Apart from these, the participants were not related. All family members were female, aged 54–77 years (mean = 65); seven were daughters and one a wife.

### Interviews

The interviews were conducted between August 2022 and May 2023 by ÅM, a female specialist nurse in elder care and PhD student with extensive competence in talking with residents and family members from her clinical experience as a nurse in elder care.

A semi-structured interview guide was developed by authors and adjusted to fit the role of the interviewee (e.g., have you/your family member experienced…). The interview guide was slightly revised and refined during the concurrent data collection and analysis to better tailor the interviews to participants with cognitive decline; the number of questions was marginally reduced and focused more directly on the experience of the proactive EoL conversation. The interview covered 19 questions, for example: “Do you recall being invited to a conversation using the conversation tool?”, “What was the conversation about?” and “How did it feel immediately after the conversation, and how does it feel now?” As the interviews progressed, it became evident that redisplaying the conversation tool helped residents who had difficulties recalling the EoL conversation, why the conversation tool was displayed in the last eight interviews with residents. The focus was on the questions in the interview guide, but the cards were showed as a reminder of the previously performed EoL conversations. On several occasions this triggered participants telling stories about EoL experiences and preferences. Interviews were audio recorded and transcribed verbatim by a professional transcription company (*n* = 15) or the first author (*n* = 3).

**Table 1:** Overview of interviews, location of interviews, duration of and time since proactive EoL conversations

	Resident interviews	Family member interviews
Number of interviews	11	7
Location of interviews		
RCH	11	1
Family member’s home		1
Telephone		5
Duration of interviews		
Min Max Median	17 min	23 min
	53 min	42 min
	39 min	33 min
Time between proactive EoL conversation and interviews		
Min Max Median	1 day	0
	61 days	71 days
	18 days	20 days

### Data analysis

Data from all interviews were included in an inductive data analysis, inspired by interpretive description [43]. The analysis was performed in five iterative phases: (1) repeated reading of the interviews, while searching for patterns and noting initial ideas for coding, which informed (2) an initial broad-based coding process, sorting and grouping data that seemed connected, using the software QSR NVivo14. This laid the ground for step (3) developing themes forming an outline for interpretation, and step (4) repeatedly reading and reviewing themes and data. This was followed by step (5) re-examination and reconstruction of each theme through a review of the initial interpretations of the data using the aim of this study as an analytic lens. The process of going back and forth between the data, codes, description, and interpretation provided new understandings of the data resulting in the three themes presented below. ÅM performed all the phases of analysis together with experienced EoL/qualitative co-authors, two women (TS, IG) and one man (LEE). TS, PhD, is a social scientist with expertise in qualitative methodology; LEE, PhD, is a health care scientist with expertise in chronic illness research; IG, PhD, is a registered nurse with expertise in palliative care and EoL research. LEE and IG have previously conducted research testing the usability of the DöBra cards conversation tool in elder care from the perspective of RCH staff [55, 56]. We used investigator triangulation to ensure credibility in the analysis. The authors held weekly meetings throughout the data collection to ensure the trustworthiness of the results. All transcripts were read several times by ÅM, while TS, LEE and IG read four interviews each, allowing discussions on alternative interpretations by the authors. Thereafter, the results were discussed at several meetings before consensus was reached about their final form.

### Ethical considerations

The study was approved by the Swedish Ethical Review Authority (ref.no. 2021–04626) and performed in accordance with the ethical standards of the 1964 Declaration of Helsinki and its later amendments [57]. All participants were provided with both verbal and written information about the purpose of the study and the details of participation and were informed that they could withdraw at any time, without explanation or consequences. All participants gave verbal and written consent to participate, including permission to audio record the interviews. Since participants with cognitive impairment participated; re-confirmation of consent and checking for verbal and non-verbal signs, in line with the MORECare-Capacity statement, were used [58]. All names used in this study have been changed and ages have been grouped to protect participants’ identities.

### Findings

**Fig. 1:**
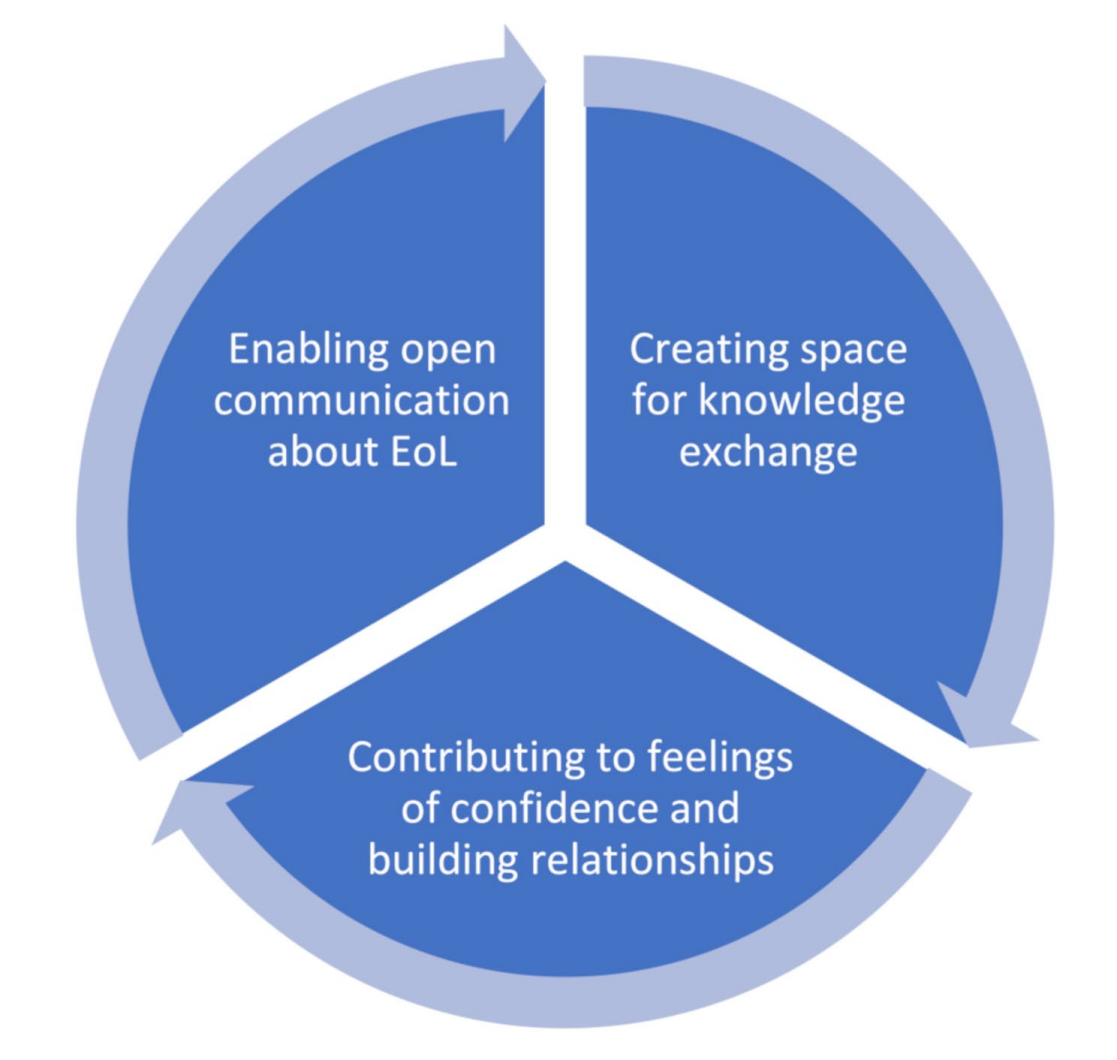
Residents and family members experienced outcomes of proactive EoL conversation presented in three interacting themes

The analysis process revealed that residents and family members experienced several outcomes of the proactive EoL conversations. This resulted in the construction of three strongly inter-related and interacting themes: Enabling open communication about EoL, Creating space for knowledge exchange, and Contributing to feelings of confidence and building relationships (see Fig. 1).

### Theme 1. Enabling open communication about EoL

Several residents described how, prior to the proactive EoL conversation, communication about EoL was generally perceived as rare and fragmented in everyday life in the RCHs. They had not discussed existential issues with staff or family members. While one resident stated that the subject of EoL could be difficult, most residents and all family members found the topic relevant in the RCH context. This was exemplified by one resident, an 80 + year-old male who welcomed the conversation:

*I thought that [conversations about EoL] was something that was part of elder care.*

*#1 resident*

Most residents expressed having had thoughts about the EoL and EoL care. One resident concluded that her approaching death was self-evident and ever-present in her daily life. Several residents mentioned previously attempting to discuss their thoughts and feelings about the final stage of their lives with staff or family members; however, they recounted examples of rejection, which they perceived as stemming from reluctance or taboo surrounding the topic. One resident, a 75 + year-old male, described:

*No, she [the staff] doesn’t want to [talk about my death]. No, she says so. She gets really frightened. Poor thing. […] Yes, she doesn’t want to hear about it.*

#18 resident

The proactive EoL conversations were welcomed as an opportunity to have an open communication about what is important to them in life. One resident, an 80 + year-old male, expressed the following:

*It became a bit more… well, more open, kind of like when we talked [during the proactive EoL conversation] you could say. […] Otherwise, it’s mostly just everyday things….*

#3 resident

As seen here, the proactive EoL conversations seem to differ from everyday small talk. Both residents and family members frequently talked about the open and inclusive nature of the proactive EoL conversation as something extraordinary in the RCH. This openness also allowed for more personal stories, beyond EoL preferences, to be communicated. These narratives involved the residents’ current and past life, including what had been important to them. There were also stories describing who the resident is as a person. This was expressed by one 55 + year-old daughter:

*We started talking in general like (…) About how he lives and functions as an individual and such. (…) How dad ended up there and the experiences he had had before ending up there. And then we maybe exchanged some personal things about our relationship.”*

*#11 family member*

As implied by this quote, the conversation format seemed to provide flexibility so that the content could vary, contributing significantly to the sense of openness. Since openness enabled the conversation to unfold according to the participants’ needs, this allowed for different subjects to arise. Family members often described how the proactive EoL conversations had been focused on the late phase of dying, the moment of passing, and post-death practicalities, including funeral arrangements. Among the recurring concerns were practices to ensure residents would not be alone when they were dying and to alleviate breathing struggles or pain. The value of communication about ease and comfort during the very last moments of a resident’s life was described by this 70 + year-old daughter:

*“But it was about dying*,* about how to make things good for mom*,* so that her death will be*,* how should I say*,* as easy as possible. And that she gets what she wants after death as well. […] What’s crucial when she’s in that situation is that the staff also know what’s most important to her. I found that extremely valuable*,* really.”*

*#14 family member*

While some residents also reflected on the final stages of life, death, and practical matters afterwards, their stories were influenced by previous experiences of death, loss, and grief. The conversation format seems, therefore, to facilitate transitions between past and present experiences, along with discussions about future EoL care preferences. This connection is exemplified by a 75 + year-old male resident:

*“Not dying*,* dying in some hospital with lots of tubes and things. I don’t want that. […] My wife lived in a residential care home during the last years of her life. But she died in the hospital*,* in the emergency room there. That wasn’t at all pleasant.”*

*#18 resident*

As seen here, when talking about his own EoL preferences, this man was clearly influenced by his previous experience of his wife’s death.

The use of the conversation tool was seen as beneficial in getting the conversations started and offering a clear framework. The flexible structure seemed particularly valuable for proactive EoL conversations involving residents with cognitive decline, as described here by a family member, 75 + year-old female, after participating in a conversation with her husband who had been diagnosed with dementia:

*“And then I think that using the cards might be a good way to have a dialogue when his memory is failing. […] I think that his mind is drifting away. […] Yes*,* that’s why I thought that it might be good to have the cards so that you can always return to the question when it drifts away.”*

*#12 family member*

Signs of cognitive decline were commonly seen during the interviews with residents, with some expressing difficulties in recalling the proactive EoL conversation, and three being unable to describe it at all. However, exposure to the conversation tool during interviews notably triggered memories and prompted discussions about EoL care preferences and past experiences. One resident, female 75 + years, with a dementia diagnosis and obvious difficulties in verbal expression, managed to verbalize her EoL care preferences -- such as being free of pain and not dying alone -- by reading the cards and engaging in discussions based on them:

*[Reading on the card ‘not to die alone’] " Well*,* that’s not good. It will be… To die alone. I don’t want to die alone. Mmm… I know that I would be*,* that I will be alone.*

*#15 resident*

This quote is one of several examples of how the written statements seemed to promote conversation and, in this case, helped the person to verbalize that she presumed that she would die alone, despite her preferences. This conversation tool may therefore be helpful in EoL conversations with people with cognitive decline.

### Theme 2. Creating space for knowledge co-creation and exchange

The proactive EoL conversations seemed to offer a space for co-creation and exchange of knowledge between residents, family members and staff. While this was more frequently described by family members, one resident, 95 + years, explicitly talked about the conversation as an opportunity to gain new perspectives:

*“Yes*,* I actually thought that the [conversation] was interesting because… [I got] to learn about other parts of life.”*

*#4 resident*

Both residents and family members described the proactive EoL conversations as an opening for exchanging knowledge about EoL values and preferences, as well as EoL practices and competence at the RCH. This facilitated the co-creation of knowledge and laid the ground for new questions, as described by this 55 + year-old daughter, after having participated with her brother in a proactive EoL conversation about her mother:

*“You got to*,* in a way*,* go through the entire situation. It also answered*,* well*,* how it will be in reality. What happens? What do you do? Can you describe how it would be? Yes*,* it led to some follow-up questions to get as concrete a picture as possible of how they do things and work. In this case*,* Amina [staff] described how they do things. It raised questions that might not have been asked otherwise or not gained a completely clear picture of before*,* if they hadn’t had this conversation.”*

*#17 family member*

When family members engaged in proactive EoL conversations together, it seemed to foster knowledge exchange between them, and to better equip them for future EoL issues. This 55 + year-old daughter, expressed it this way:

*“Mum hasn’t said so much herself*,* and she can’t say much herself about things anymore. I think both of us thought it’s probably good that we have such a conversation. And have it together. So that we are a bit prepared and in agreement. That’s also important when you can’t get information directly from the person it concerns.”*

*#17 family member*

As seen here, the space created had provided an opportunity to co-create knowledge forming a mutual understanding about EoL care in the RCH. This was said to be a measure to prevent potential conflicts between family members, and between family members and staff, in the future. Several participants considered it important to share this new knowledge with others; some had already talked to other family members. Both residents and family members reported expectations that the staff would share knowledge about residents’ EoL care preferences in the care team. Several family members expressed feeling a sense of responsibility to communicate the residents’ values and preferences, especially when the residents themselves had difficulty communicating these due to cognitive impairment. Acting as surrogates was considered both challenging and necessary– as a means of safeguarding the residents’ autonomy::

*“Yeah*,* it’s difficult*,* and it kind of makes you feel quite humble facing the situation*,* somewhere*,* and sensitive. And it becomes even more important that it’s done right*,* that you have that focus. But I feel that we had that. But*,* absolutely*,* it’s really difficult. If you think: how do you do her justice?”*

*#17 family member*

Family members were not always confident about the residents’ EoL preferences, but knowing the person well, and having known them for a long time, made it easier, helping them in their role, as articulated by a 55 + year-old daughter:

*“I am their spokesperson. I know mum and dad very well. We’ve had a very good relationship in recent years. I’ve been really involved with them throughout their dementia journey. Right from the beginning and as it progressed. So*,* I felt like I had them in my thoughts the whole time [during the proactive EoL conversation].*

*#11 family member*

Several family members expressed uncertainty about including residents in the conversations. Their arguments were mainly related to concerns about the conversations evoking negative feelings of sadness, depression, or anxiety. Another argument was the belief that residents lacked interest in discussing the subject. The most prevalent argument was uncertainties about the resident’s capability to communicate and use the conversation tool, due to cognitive decline.

### Theme 3. Building relationships and feelings of confidence

Several family members expressed how being invited to participate in a proactive EoL conversation with staff conveyed understanding, empathy, and a recognition of the resident’s EoL care needs. Acknowledging the resident’s status as being in a late stage in life and providing an opportunity to reflect and openly discuss this, created a basis for feeling reassured, here described by one daughter, 70 + years old:

*“Basically*,* the feelings it evoked were like*,* ‘Here are staff members who care.’ I mean*,* here*,* they care*,* they know that death is coming. She won’t get better*,* she won’t leave there*,* it’s the only way out*,* and they care. They care about her*,* they care about doing the best possible for her*,* and that*,* I feel*,* is reassuring.”*

*#14 family member*

The proactive EoL conversations indicated that staff care about the resident, which was pivotal for the family member’s feelings of confidence, and created conditions for building relationships, both between the staff and residents and the staff and family members. These relationships were characterized by a shared and genuine commitment to the resident’s wellbeing. While several residents emphasized their relationships with family members, some also described their relationships with staff as essential to meeting their needs in daily life, this sentiment was expressed by one resident, a 75 + year-old male:

*“I feel that those who work here are my friends. They are the ones I usually have around me.”*.

*#18 resident*

This statement suggests that staff may play a significant role in the residents’ daily lives, underscoring the value of being known as the person you are. Both residents and family members described how knowing the person was fundamental for building relationships and that the proactive EoL conversations provided a rare opportunity for this. During the interviews, none of the participants suggested it was necessary to have established a professional relationship before engaging in proactive EoL conversations. Instead, these conversations appeared to foster the building of relationships between those participating.

Family members described how they experienced the proactive EoL conversations as an invitation to be involved in the resident’s care, and thereby being recognized as an important person in the resident’s life. This contributed to a sense of confidence and trust in the staffs’ intentions to provide the best possible care. One daughter, 60 + years, expressed how the proactive EoL conversations contributed to trust:

*“However*,* if I wasn’t there when mum and dad pass away*,* and Marianne [staff] was*,* I would feel very*,* very reassured. She was extraordinary and so understanding during the conversation. […] She felt professional in a way*,* not giving her own opinions but showing enormous empathy.”*

*#10 family member*

For this person, it appears that the staff’s ability to listen attentively had positively influenced her trust in the person/staff she had the conversation with, promoting feelings of confidence and of being understood. Trust was also said to facilitate future communication, as expressed by another family member:

*“…this makes it feel easier to turn to her again*,* if there’s anything. So*,* we got to know each other a bit more then.”*

*#14 family member.*

## Discussion

We used an interpretive description approach to explore the outcomes experienced by residents and family members who had participated in proactive EoL conversations at RCHs. We identified three interconnected themes: Enabling open communication about EoL, creating space for knowledge co-creation and exchange, and building relationships and feelings of confidence (Fig. 1). The themes were understood to interact and influence each other, e.g., that opportunities for open communication concerning EoL foster knowledge exchange over time, which in turn can improve relationships between those present in the conversation. This may consequently improve conditions for further EoL communication among those involved.

In this study, we found that the use of a conversation tool offered a supportive and flexible approach to enable open communication, allowing residents and family members to express their thoughts and preferences about the EoL. The findings suggest that people with varying cognitive function may also benefit from EoL conversations in this format. Despite a widely accepted need for EoL conversations with people with cognitive decline early in the care trajectory [29, 59], such communication is often performed too late, prohibiting residents’ participation [60]. In light of this, we suggest that proactive EoL conversations be regarded as a process, with repeated conversations as the person’s health deteriorates. We found, as did Saevareid et al. [61], that people with cognitive decline can benefit from EoL conversations and provide essential information about their EoL care values and preferences. Furthermore, being listened to and being seen as a unique person has been found to be fundamental in contributing to existential wellbeing, regardless of a resident’s present experience of independence, activeness, and autonomy [62]. Our findings argue against routinely excluding people with cognitive decline. By using this inclusive and flexible tool, staff and family members can feel confident to include people with cognitive decline in conversations about their EoL values and preferences and thereby involve them in planning their own care. However, special consideration may need to be given to people with cognitive difficulties and it may be helpful to schedule conversations at an appropriate time in an undisturbed place and ensure that conditions for communication are as good as possible. The statements on the cards promoted thoughts and verbal reflection about the past, present, and future, and therefore did not only focus on care at the EoL, but rather life as a whole. It is, therefore, also suggested that EoL communication with older adults encompasses their current life [63] and their preferences concerning living well in the present [64]. Other studies have reported that older adults often live one day at a time [32], and think about the past [65] rather than an uncertain future [64]. This means that the EoL conversations, with their focus on what is important to the resident, need to be open to including these aspects. There is no “one size fits all”; instead, a flexible and adaptive conversation format is called for. Structured yet open communication may therefore foster the reflection and discussions that are needed to prepare for informed EoL care decisions, which communication focusing only on treatment options and hospital admission may miss [66]. While previous research clearly shows that communication about EoL can be perceived and received in different ways, and that different reactions may occur [32], we found that both residents and family members welcomed reflecting on EoL issues [67]. It is worth noting that staff competence and preparation is an important component of communication in RCHs [68], since staff have the ability to ensure that these conversations are tailored to participants’ needs. To provide open conversations, a conversation tool with less of a “checklist” approach, as used in this study, is helpful in triggering reflections about a topic that may be perceived as difficult in a context that has been described as “task-oriented”.

Proactive EoL conversations were found to initiate a process of knowledge exchange which may contribute to a shared understanding among those involved. Our results indicate that experiential and professional knowledge was shared and valued, which may prevent potential current and future misunderstandings and disagreements. Knowledge exchange has been defined as a dynamic and fluid process of learning and sharing, incorporating distinct forms of knowledge from multiple sources [69], contrasting with communication involving a more traditional information transfer not uncommonly used in healthcare [70, 71]. Such knowledge exchange processes have been reported to result in improved outcomes, e.g., building relationships or bonds between residents, family members, and staff, being ‘‘known’’ by the staff, and having preferences honored [72]. The underlying goal of proactive EoL conversations is to allow for the narratives of residents to be heard, which is also a key to establishing person centred care as described by Ekman et al. [73] who suggest that sharing experiences and exchanging knowledge is a way to build partnerships. Partnerships in turn are a prerequisite for person-centered care, suggested as being particularly important in older populations [6–8]. As noted by Thoresen et al. [66], the purpose of the EoL conversations, and the persons they are intended for, need to be considered. In addition to promoting knowledge exchange, EoL conversations in this format may promote family involvement by strengthening family-staff relations, which has been highlighted as imperative for residents’ and family members’ wellbeing [74]. It has previously been argued that EoL communication can help family members be prepared for what lies ahead, and that this is advantageous to those who often feel responsible for assisting in decision-making [75]. This was also visible in our findings, where the family members’ concern for their loved ones in the RCHs was noticeable throughout the interviews and analysis process. We suggest, therefore, that EoL conversations in this form may be a means of supporting family members, since they have the potential to promote a person-centered dialogue, revealing underlying values and preferences, thereby contributing to feelings of confidence, and building relationships. This has also been pointed out as essential for people living with life-limiting illness, who reported that feelings of confidence and maintaining their identity were closely associated with relationships with family members, social networks, and staff [76]. Building and maintaining relationships is thus prioritized by persons living under these circumstances [76].

From previous research we know that the conversation format used in this study has been shown to promote reflections on EoL care values and preferences from the perspectives of community dwelling older adults without known cognitive impairments [77, 78] and of RCH staff [56]. In the present study we found that proactive EoL conversations have the potential to contribute to what other studies have reported as being high quality EoL care in RCHs [79]. This includes providing emotional and psychosocial support; becoming informed; promoting family understanding; and establishing a partnership with family carers by involving and guiding them in shared decision-making [80]. However, we found, as did Sussman et al. [81], that family members felt a need to protect their loved ones with dementia from what may be perceived as a potentially upsetting subject. This highlights the importance of exploring resident/family relationship patterns [72] and sensitively offering proactive EoL conversations, but at the same time being aware that they do not suit everyone.

Nevertheless, the hesitance staff members to initiate conversations about EoL in RCHs remains and several barriers/challenges have been identified [82]. One such barrier/challenge is the general need for building broader competence regarding EoL communication and care as a public health issue, which is of importance beyond institutions and involves us all [83, 84]. A suggestion for addressing this is the process of reflecting jointly on EoL care issues. Death literacy is a term used to describe this acquired EoL care competence [85], a concept suggesting that EoL competence is developed through experiential learning and which supports people’s readiness for engaging in EoL conversations [86]. This relatively new concept has been explored in the Swedish context [87, 88], where levels of knowledge about palliative care have been described as somewhat low among the general public [89]. Further research should explore if proactive EoL conversations could contribute to the development of death literacy among residents, family members, and staff.

### Strength and limitations

This study has both limitations and strengths. Despite wide recognition of the need to implement adapted formats of EoL communication in RCHs, there are still only a few examples of how this is experienced in the clinical context. In this study, a research-based conversation tool was used to promote EoL conversations between staff and residents and/or family members. We aimed to include both residents and family members, and analyze the combined data, as they were viewed as having complementary and interwoven experiences. We did not specifically exclude people with cognitive decline; instead, we used the MORECare-Capacity statement solutions which include consulting healthcare staff and using a tailored and iterative consent process [58]. However, one weakness of the study is the homogeneity of the invited participants. Most of the participants were women. This is in line with literature indicating that women are more likely to use elder care services [49], as well as being the most common caregivers [56,57]. In addition, only two participants were born outside of Sweden, where one was born outside of Europe. It is also worth noting that this study was conducted in five non-profit RCHs in one urban municipality in Sweden, where municipal self-government is a fundamental principle and local variations are well known [90]. This means that the results of the study cannot automatically be applied to any RCH context, which is a weakness that must be considered concerning transferability. It is also worth considering that participants had mostly positive experiences of the proactive EoL conversations, which may be explained by the recruitment strategy, i.e., the staff inviting individuals who they felt comfortable having the conversations with. The time that had elapsed between the EoL conversations and the interviews was long in some cases, with a median of 18–20 days. This might have affected the participants’ views of the conversations, in particular participants with cognitive decline. However, we have considered the procedure and argue that the findings can still contribute to the ongoing efforts to explore EoL conversations in RCHs.

## Conclusions

This study provides insights into the outcomes of proactive EoL conversations in RCHs as experienced by residents and family members. Proactive EoL conversations in this format were found to generate several beneficial outcomes for residents and family members, including those with cognitive decline.

We conclude that proactive EoL conversations allowed the narratives of residents and family members to be heard, and that open communication about EoL care may foster knowledge exchange over time. This in turn may aid feelings of confidence and improved relationships between those present in the conversation. Proactive EoL conversations may therefore strengthen person-centered care and family support in this context. Based on these findings, proactive EoL conversations have the potential to be used by RCH staff.

## Electronic supplementary material

Below is the link to the electronic supplementary material.

Supplementary Material 1

## Data Availability

The data generated and analyzed during the current study are not available for public use for confidentiality reasons but are available from the corresponding author on reasonable request.

## Footnotes

1 In Swedish, *DöBra* is a play on words, literally meaning ’Dying Well’, but also an idiom roughly equivalent to ’awesome’ in English.

## Abbreviations

RCH: Residential Care Home
EoL: end-of-life
ACP: Advance Care Planning
