# Supplementary material for: Proactive end-of-life conversations in residential care homes: a qualitative interview study exploring residents’ and family members’ experiences
Source: BMC Geriatr. 2025 Apr 25;25:279. doi: 10.1186/s12877-025-05916-7 (PMC12032711; doi:10.1186/s12877-025-05916-7)
Supplement: Supplementary file 1 — Supplementary Material 1 [file 12877_2025_5916_MOESM1_ESM.docx]

Supplement file 1. Analysis process and theme development. Matrix contains examples of quotes, broad based coding, description, and themes.

| **Data** | **Broad based coding** | **Description** | **Initial interpretation** | **Theme** |
| --- | --- | --- | --- | --- |
| *"Not dying, dying in some hospital with lots of tubes and things. I don’t want that. [...] My wife lived in a residential care home during the last years of her life. But she died in the hospital, in the emergency room there. That wasn’t at all pleasant."* | EoL conversations trigger stories about individual experiences | EoL conversations trigger stories about individual experiences that influence own reflections about EoL and care preferences | Open communication allows stories about previous experiences of EoL as a way to communicate own EoL values and preferences | 1. Enabling open communication about EoL |
| *"You got to, in a way, go through the entire situation. It also answered, well, how it will be in reality. What happens? What do you do? Can you describe how it would be? Yes, it led to some follow-up questions to get as concrete a picture as possible of how they do things and work. In this case, Amina [staff] described how they do things. It raised questions that might not have been asked otherwise or not gained a completely clear picture of before, if they hadn’t had this conversation."* | EoL conversations is an opportunity for asking question and learning about EoL care | EoL conversations provide space for new knowledge adapted to participants needs and this may create and a clearer picture for family members. | Open communication creates opportunity for participants to create and exchange new knowledge about various aspects of EoL care in the NH. | 2. Creating space for knowledge co-creation and exchange |
| *"However, if I wasn’t there when mum and dad pass away, and Marianne [staff] was, I would feel very, very reassured. She was extraordinary and so understanding during the conversation. [...] She felt professional in a way, not giving her own opinions but showing enormous empathy."* | EoL conversations as an opportunity to communicate EoL issues | EoL conversations enables staff to communicate empathy and understanding through being professional and responsive and this builds feelings of safety and trust among family members. | EoL converations is an opportunity to create trusting relationship between staff and family member/resident | 3.Building relationships and feelings of confidence |
